# Obstructive Sleep Apnea in Adults and Ear, Nose, and Throat (ENT) Health: A Narrative Review

**DOI:** 10.7759/cureus.47637

**Published:** 2023-10-25

**Authors:** Nishtha Choudhury, Prasad Deshmukh

**Affiliations:** 1 Ear Nose and Throat, Jawaharlal Nehru Medical College, Datta Meghe Institute of Higher Education and Research, Wardha, IND

**Keywords:** multi-layer surgeries, upper airway, continuous positive airway pressure (cpap), polysomnography, obstructive sleep apnea (osa)

## Abstract

Obstructive sleep apnea (OSA), a form of sleep-disordered breathing, is a significant health concern that leads to substantial morbidity. The collapse or occlusion of the upper airway, which results in reduced or cessation of airflow, is the pathophysiology of sleep apnea. The condition has been attributed to numerous cardiovascular, metabolic, and neuropsychological issues and carries serious health concerns. The ensuing intermittent hypoxia and sleep disruption set off a chain of physiological reactions that aid in developing endothelial dysfunction, systemic inflammation, and oxidative stress. The following line of treatment depends on the appropriate diagnosis of sleep apnea and the underlying cause. The gold standard for diagnosis is polysomnography (PSG), which assesses different physiological parameters during sleep. However, because polysomnography is expensive, patients may use more friendly screening and diagnostic testing kits, like home sleep apnea testing. The clinical symptoms and head and neck history may reveal essential risk factors. The primary objectives of management treatments for sleep apnea are to lessen symptoms, enhance sleep quality, and reduce any health concerns that may be present. It is advised to start with lifestyle changes such as quitting alcohol and sedative use, losing weight, and exercising frequently. The primary treatment for moderate to severe sleep apnea is continuous positive airway pressure (CPAP) therapy, which includes administering pressurized air to keep the airway open while you sleep. Oral appliances, positional therapy, surgery, and complementary therapies are other treatment choices that can be adapted to each patient's needs and preferences. The goal of the review is to evaluate the morphological and functional aspects of the upper airway, including the nose and throat, that influence the onset and severity of OSA. With a focus on the interaction between otorhinolaryngologists, sleep medicine specialists, and other healthcare professionals, we aim to consider how OSA affects otorhinolaryngology-related medical issues, look at any potential reciprocal relationships, and provide a summary of the interdisciplinary management strategy for OSA. We tried to analyse the various surgical and non-surgical therapy options for OSA management available in the otorhinolaryngology field for improving OSA symptoms and results.

## Introduction and background

Only one-fourth of patients with obstructive sleep apnea (OSA) in the community report experiencing this symptom, despite it being the most prevalent OSA-presenting indication. It has been demonstrated that OSA is linked to a two- to three-fold increased risk of metabolic and cardiovascular conditions. Home sleep apnea testing can detect OSA in people about 80% of the time. Diet and exercise, positive pressure ventilation, dental appliances that propel the jaw outward while the person is asleep, and surgically widening the upper respiratory tract by altering the pharyngeal soft tissues or facial skeleton are all effective treatments. Hypoglossal nerve stimulation benefits specific individuals with central sleep disturbance who have a BMI under 32. There are few fruitful pharmacological treatment options [1].

The following variables were examined in a study: minimum blood oxygen saturation, respiratory disturbance index (RDI), absolute and recorded fluctuations in RDI, supine and non-supine NREM and REM RDI, and rapid eye movement (REM) RDI. There were estimated connections between treatment response and a mandibular advancement splint (MAS). Patients who, at baseline, had more severe airway rigidity and OSA benefited more with MAS therapy. Furthermore, treatment was more effective for airway obstruction brought on by craniofacial abnormalities than soft tissue obstruction [2].

Frequently, breathing pauses while you sleep are a common symptom of sleep apnea. Your body may, as a result, not receive enough oxygen. If someone claims to gasp or snore while they sleep or feels other signs of poor sleep, such as increased daily exhaustion, they may wish to consult a doctor about sleep apnea. Sleep apnea can come in two different forms. Obstructive sleep apnea occurs when the upper airway is perpetually closed as individuals sleep, reducing or stopping airflow. The most common type of sleep apnea is this one. Large tonsils, hormonal changes, and obesity can all restrict the airway and raise the chance of developing obstructive sleep apnea. Another form, the central one, hinders the brain from processing and sending the signals required for you to fall asleep. Sleep apnea comes in two primary varieties. When the upper respiratory tract is frequently closed while a person is sleeping, either less airflow occurs or airflow stops entirely, and obstructive sleep apnea develops. The majority of cases of sleep apnea are of this type. Large tonsils, endocrinal changes, and obesity all contribute to the narrowing of the respiratory tract and increased risk of obstructive sleep apnea [3].

## Review

Methodology

**Figure 1:**
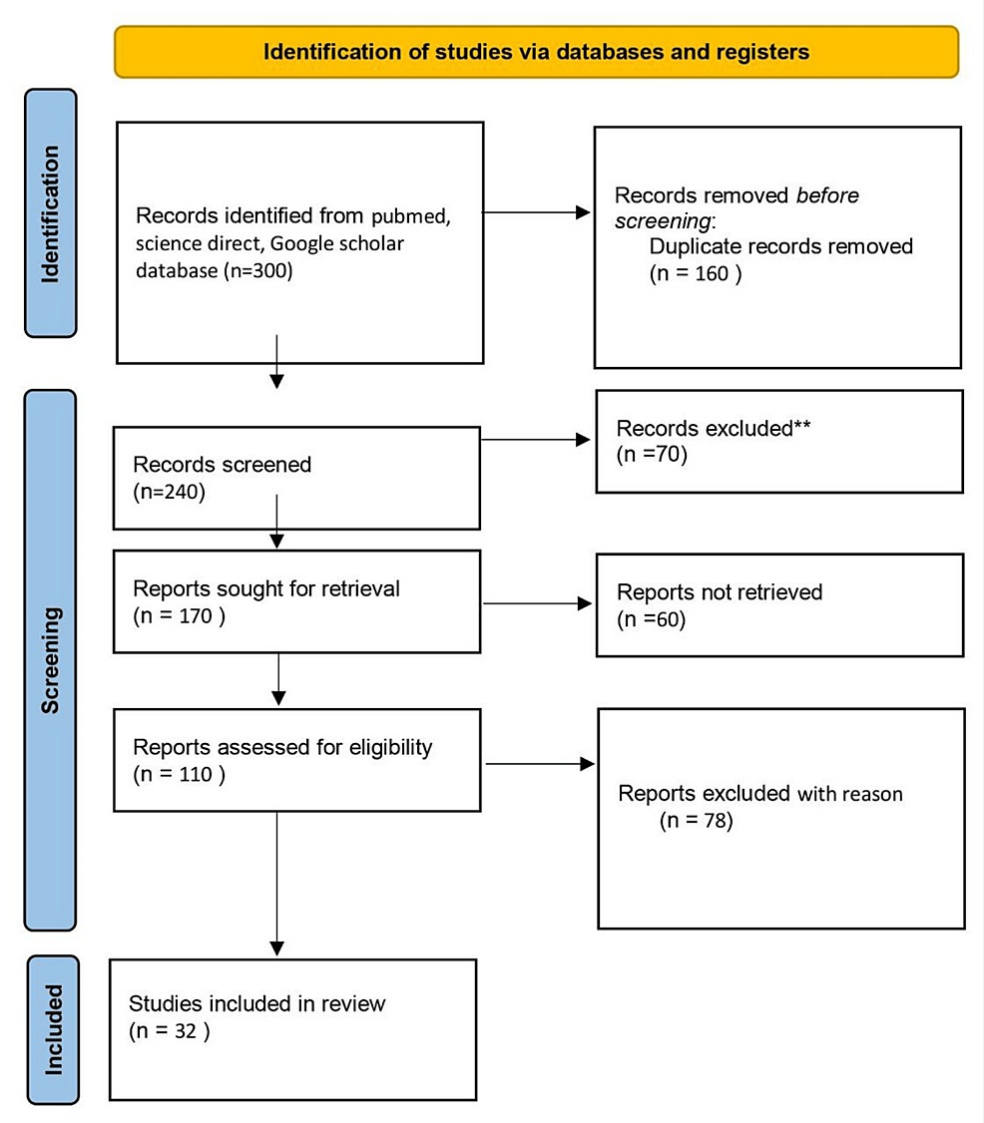
PRISMA flow chart for literature search. Adopted from the Preferred Reporting Items for Systematic Reviews and Meta-Analyses (PRISMA).

We undertook a search through PubMed and CENTRAL in March 2023 using keywords such as “obstructive sleep apnea” and "ENT" (((obstructive sleep apnea [Title/Abstract]) OR (OSA [Title/Abstract])) OR (continuous positive airway pressure [Title/Abstract])) OR ("CPAP" [MeSH Terms]) AND (("quality of life" [Title/Abstract]) OR (QoL [Title/Abstract])) OR ("quality of life" [MeSH Terms]). We additionally searched for key references in the bibliographies of the relevant studies. The search was updated in July 2023. One reviewer independently monitored the retrieved studies against the inclusion criteria in the beginning, based on the title and abstract, and then on full texts. Another reviewer also reviewed approximately 20% of these studies to validate their inclusion. Differences were resolved through discussion. One reviewer extracted the data from studies according to the mental, physical, and social health aspects of health-related quality of life (HRQOL). Data were extracted and tabulated based on details about the study design, population, quality of life (QoL) instrument used, QoL domain assessed, and study findings. We included studies that assessed the effect of obstructive sleep apnea on QoL both during and after treatment, irrespective of the study design, geographic location, age, gender, type of OSA, or type of treatment. We excluded studies other than OSA if full-text links were unavailable to the reviewers. Figure 1 shows the PRISMA flow chart for the literature search.

Prevalence

In a cohort study, 602 working individuals between 30 and 60 had an average amount of apnea and hypopnea episodes per hour of sleep, also known as one's apnea-hypopnea score. The patients with sleep apnea/hypopnea at scores of 5, 10, and 15 have been estimated from the cohort. Compared to men aged 30 to 39, men between 40 and 49 showed considerably greater frequencies at all three levels. The only statistically significant difference between the women sections was the probability of having an apnea-hypopnea score of five or more, greater among older women (aged 50 to 60) than in younger women. Males seemed to experience OSA more than females in all age groups and at all apnea-hypopnea cutoff points; all differences were significantly different, with the notable exclusion of patients aged 30 to 39 with apnea-hypopnea scores of 15 or higher [4]. Both study measures used actual samples drawn from the general public and were wrapped up using data obtained from another cross-sectional survey. In a Spanish study of 2148 people aged 30-70, sleep-disordered breathing was 26.2% in men and 28.0% in women. Most subjects were investigated using recordings with fewer channels and without directly tracking airflow. Three hundred ninety individuals, a relatively small sample, had complete polysomnography (PSG). In a Brazilian research article for a study with 1042 individuals, ages 20 to 80, the recently engineered breathing sensors were used to track variations in nasal pressure. According to the article, it was estimated that 30% to 40% of men and women have a high disease occurrence rate [5].

Pathophysiology

The respiratory tract has been dramatically affected by upper airway collapse brought on by obesity, craniofacial anatomical flaws, and soft tissue changes. All people with obstructive sleep apnea syndrome (OSAS) have different levels of upper airway structural damage. Peripheral pressure increases when one lies down at night because the fluid collected in the legs all day moves to the upper torso. A nocturnal rostral fluid shift is what is happening here. Most patients also have mucosal oedema, which is uncertain in its cause. When you are awake, your brain activity keeps the muscles in your throat firm, protecting the throat from collapsing. If this muscle is not engaged during REM sleep, the airway could collapse (chemosensitivity, central respiratory neurons, and ventilatory drive). Hypoxia-inducible factor -1 (HIF-1), a potent regulator for oxygen hemostasis, has a different function in various oxygen environments. Normal HIF-1 synthesis occurs in the nucleus, where it is translated into a protein in the cytoplasm. PHD is then responsible for hydroxylating the protein. The Von Hippel-Lindau (VHL) protein is connected to it and then degraded by ubiquitination. Under typical conditions, hypoxia forms there, links up with HIF-1, and then joins forces with p300/CBP on the hypoxia response elements to start the quality record. Over time, hypoxia stops the deterioration of HIF-1. In contrast to continuous hypoxia or normal oxygen levels, KDM4A, KDM4B, and KDM4C showed higher activity. Due to this increased activity, the HIF1 gene's H3K9me3 demethylation occurred at a higher rate. As a result, HIF1 mRNA is generated more frequently. A surge in intracellular ROS brought on by intermittent hypoxia can activate PLC and lead it to create IP3 and DAG. These two messengers, in turn, stimulate transcriptional activity and the production of the HIF-1 protein via intracellular signal transduction pathways. In OSAS, there are additional IH-induced signalling pathways. IH uses some techniques to keep the PAI-1 record up-to-date [6].

OSA in relation to ear, nose, and throat

**Figure 2:**
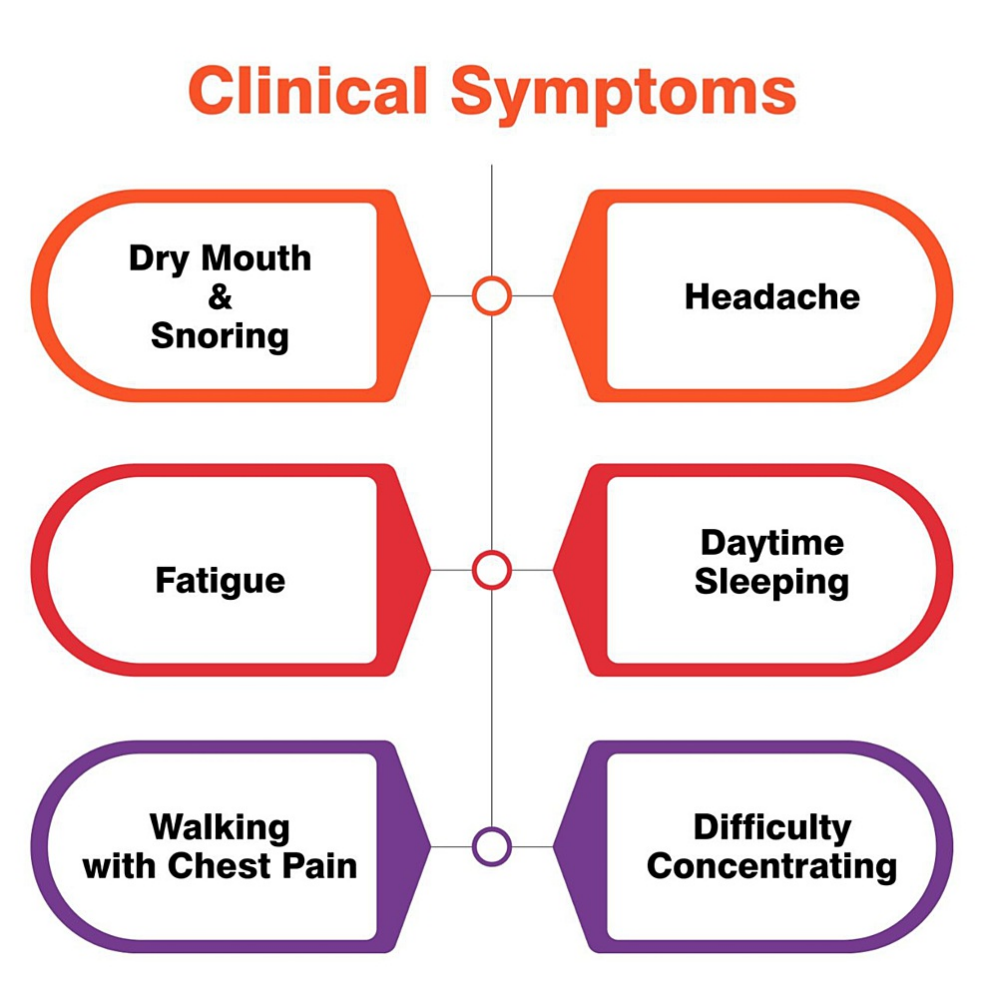
Symptoms of sleep apnea. Self created.

Figure 2 shows some common symptoms of sleep apnea.

Relation to the Eustachian Tube Dysfunction

Numerous studies have explored possible connections between OSAS and the physiological mechanism of the Eustachian tube. Young individuals with OSA are more likely to have Eustachian tube dysfunction (ETD) due to two distinct clinical manifestations of a shared structural problem, such as adenoid enlargement. Adenoid hypertrophy and adenoiditis can cause the nasopharynx to become functionally obstructed, which can cause OSA and Eustachian tube dysfunction due to nasal airway obstruction and Eustachian tube inflammation, respectively [7]. Growing OSA severity among youngsters is linked to negative middle ear pressure (MEP), which raises the possibility that OSA-related negative pressure is conveyed to the middle ear. People with more severe OSA have higher positive MEPs [8]. Upper airway resistance in obstructive sleep apnea syndrome sufferers can impact middle ear pressure. Mild, moderate, and severe OSAS do not hinder middle ear pressure; however, severe OSAS may cause a rise in the (negative) middle ear pressure. Long-term continuous positive airway pressure therapy may return middle ear pressure to normal in patients with severe otosclerosis [9].

Relation to Nose and Upper Respiratory Tract

Fifteen percent of the overall population claims to have nasal blockages. Decreased nasal patency can be caused by a wide range of anatomical issues, including septal deviation, larger turbinates, and nasal valve collapse. Additionally, allergic and non-allergic rhinitis, chronic rhinosinusitis alongside or excluding nasal polyposis, and an array of inflammatory illnesses of the nasal mucosa can also result in nasal blockage. More than 33% of OSA patients exhibited nasal obstruction at night. The least cross-sectional area of each of the nasal valves was, on average, lesser in patients with nocturnal nasal blockage. Likewise, nasal blockage was associated with slightly lower measures of daytime drowsiness, late insomnia, and mental quality of life [10]. Prior to falling asleep, all participants who gave consent took a standard nasal examination by a researcher and completed questionnaires for the Epworth Sleepiness Scale (ESS), Snore Outcomes Survey (SOS), and Nasal Obstruction Symptom Evaluation (NOSE). Turbinate hypertrophy and the NOSE score were found to be correlated (0.357), whereas external and internal nasal valve collapse have been demonstrated to be correlated (0.4986) [11].

Both awake examinations and drug-induced sleep endoscopy in patients with obstructive sleep apnea showed that the epiglottis was mainly of a linear shape and did not have the distinctive anterior convexity in its upper half in more than 60% of patients with obstruction at the epiglottis or tongue base. The epiglottis has deformed due to the degeneration of the suspensory apparatus, which holds it in place and maintains its standard shape. This would make it easier to pick out patients with this degree of restriction, which might ultimately lead to more appropriate decisions about the best course of treatment [12]. When it comes to respiratory problems in OSAS, tongue-base-associated obstructions (TBOs) are more damaging than soft palate-associated obstructions (SPOs). The average duration of apnea and hypopnea, along with the proportion of time spent snoring, accurately predicted the TBO percentage. Compared to SPOs, TBOs had significantly longer mean apnea periods, a higher percentage of apneas than apnea-hypopneas, mean O2 desaturation, and a higher proportion of event-related discomforts [13]. Thirty-one patients with severe OSA who underwent modified tongue base suspension (mTBS) were included in a case study with organized data collection. Prior to surgery, each patient underwent PSG and continuous positive airway pressure (CPAP) titration on two separate nights. After surgery, at the end of the half-year of recuperation, patients had a control PSG and CPAP titration. The procedure was regarded as successful when the mean AHI decreased by 50% and the AHI dropped to 20/h. Overall, 24 patients (or 77.4%) excelled the criteria for successful surgery. The mTBS is a safe and practical method that improves CPAP dosages in patients with severe OSA [14].

Both non-invasive and invasive nasal treatments improve sleep quality significantly, making it possible for patients to experience more restorative sleep that improves many areas of their quality of life. However, the effect of both conservative and surgical nasal therapies is typically negligible when assessing the grimness of OSA using the apnea-hypopnea index, including just a few unusual cases demonstrating notable improvements. However, preliminary evidence suggests that effective nasal surgery might make nasal ventilation therapy more practical by lowering the necessary pressure levels [15]. Intermittent breathing cessation can be partial or total, though the latter is less common. Comparing these patients to people of comparable age who do not have these traits, analysis of the sleep electroencephalogram reveals that these patients typically struggle to enter deep sleep and display lower levels of stage 1 REM sleep. They suffer from chronic sleep deprivation as a result, which causes daytime sleepiness, chronic exhaustion, and frequent personality disorders such as paranoia, agitated depression, and anger. Monitoring while you sleep is required to provide a firm diagnosis of this syndrome. This monitoring must include electroencephalogram analysis, measurement of belly movement, and thermocouple assessment of nasal airflow [16].

The symptoms of gastroesophageal reflux disease (GERD), which may be a potential aggravating factor in causing esophageal cancer, include coughing due to reflux and apnea. Characterized by the Eustachian tube's inability to drain the middle ear's secretions, ETD maintains the air pressure of the middle ear and protects the ear from infections. According to a Korean study, patients with GERD are more likely to have OSA than the benchmark group, and this association is free of other factors. According to this study, GERD significantly adds to patients with OSA having deteriorating ETS-7 and ETD-Q scores (eustachian tube score and eustachian tube dysfunction questionnaire) results. The greater risk of ETD in OSA patients may be due to the high frequency of GERD in this group of people [17].

OSA due to other causes

Obesity

In obese patients, excessive fat deposition at two separate anatomical locations may result in OSA. An excessive amount of adipose tissue in the maxillomandibular junction may arouse tissue pressure and narrow the pharyngeal airway. Progressing Tacheal traction forces are primarily to blame for the lung volume relationship of pharyngeal airway patency. Leptin and other hormones and cytokines associated with obesity may interfere with the normal respiratory rate, increasing the progression of obstructive sleep apnea. The presence of soft tissue deposition in the submandibular region serves as a sign of anatomical imbalance and is treated as such. Excessive central adipose tissue buildup may result in decreased lung function and longitudinal pharyngeal wall stress [18].

The morphology of the upper airway mesenchymal tissues and the cervical-craniofacial skeletal system was comprehensively examined. Both OSA groups showed modifications in the morphology of the cervical-craniofacial skeletal structure and the soft tissues of the upper respiratory tract as compared to the controls. The only anatomical abnormalities in the healthy OSA patients were the cervical-craniofacial skeletal structures, while in the overweighing OSA patients, the hyoid bone location, head posture, and upper respiratory pathway soft tissue morphology were more out of the ordinary. The findings suggest that the two groups of OSA patients require unique treatment regimens. Cephalometric analysis, the apparatus for calculating BMI, is strongly advised in conjunction with various BMI components. Cephalometric examination, in conjunction with other BMI issues, is an important technique for identifying and deciding treatment for OSA patients [19].

*Cardiovascular Relations* 

Paroxysmal nocturnal dyspnea (PND) is a typical symptom in people with acute decompensated heart failure (ADHF). PND and some sleep apnea (SA) symptoms may be related to the nightly deterioration of hemodynamics in failing hearts. It could mean that SA could increase the risk of PND in people with ADHF when combined with nocturnal hemodynamic decline [20]. Sleep apnea accelerates chronic heart failure and may be a sign of a poor prognosis. It is described by a pathologic breathing pause lasting longer than 10 seconds while you sleep. It actually causes a range of mechanical, hemodynamic, chemical, and inflammatory changes that deteriorate the cardiovascular homeostasis of patients with heart failure. A loss of respiration and a lack of respiratory effort cause breathing disruptions in about 40% to 60% of heart failure patients. Patients with OSA stop breathing even though they are trying to breathe when their throat muscles relax. This kind only affects about 3% of the general population; lifestyle modifications pose a potential treatment [21].

Diagnosis and management

Results With Physical Examination of Head and Neck

As demonstrated in a group of patients who sought medical attention for obstructive sleep apnea-hypopnea syndrome at a public otorhinolaryngology institution. Each patient undertook a complete evaluation, which entailed a clinical history, an otolaryngological exam, and an assessment of a drowsiness scale. The physical examination encompasses pharyngeal soft tissue inspection, anterior rhinoscopy, and evaluation of facial skeletal growth. The occurrence and depth of obstructive sleep apnea-hypopnea syndrome appear to be correlated with a number of elements, including BMI, the new Mallampati classification, and abnormalities of the pharynx. In a very small number of cases, the tonsils were swollen. Additionally, nasal obstruction symptoms were frequently seen in sleep apnea patients [22]. Taking into account both skeletal and soft tissue anomalies, the physical examination of the head and neck revealed major disparities between OSA and non-apneic patients. Non-apneic patients had a larger turbinate and less pronounced septal deviation [23].

Pharmacological Approach

Patients with chronic allergic rhinitis (PAR) frequently experience nasal congestion, sleep disturbances, fatigue during the day, and somnolence. Budesonide (BUD), an efficient intranasal steroid used as a topical anti-inflammatory, will reduce nasal congestion and improve the patient's quality of life [24]. The doctor may choose the following line of treatment, which might be helpful for the patient. Table 1 shows some important treatment options and advancements.

**Table 1:** The treatment options. Self created. CPAP: continuous positive airway pressure, NSAIDS: nonsteroidal anti-inflammatory drugs.

Non-surgical	Surgical
CPAP	Tracheostomy
NSAIDS	Uvulopalatopharyngoplasty
Anti-allergics	Radiofrequency ablation
Mandibular advancement devices	Palatal implants
Nasal dilators	Multi-layer surgeries

Non-surgical Approach

It is possible to employ mandibular advancement devices (MADs) instead of nasal CPAP therapy. A self-adjustable intraoral sleep apnea device (ISAD) that enables three-dimensional movement of the lower jaw, however, may be effective in some situations [25]. After 30 months, 56-68% of patients are still using oral appliances, according to a summary of follow-up compliance data. The most common negative effects are sore teeth and excessive salivation. Effectiveness and adverse effects are influenced by the appliance's type, degree of protrusion, vertical opening, and other factors. Oral appliances do contribute to the control of snoring and sleep apnea, although they are less effective than CPAP [26]. A recently introduced internal nasal dilator could dramatically enhance respiratory results and sleep quality [27]. MAD, which are customized, have demonstrated improved treatment potency in dealing with regular snoring and OSA when connected with morphological changes discovered by radiological tests [28].

Surgical Approach

A tracheostomy is a tried and true treatment for OSA, but it should only be performed when all other options are either ineffective or impracticable. The apnea-hypopnea index may not always return to normal in those who have uvulopalatopharyngoplasty (UPPP). Oral appliances or positive airway pressure therapy are among the initial choices for taking care of patients with average OSA. Patients whose UPPP has failed may opt for bariatric or multi-level surgeries and have numerous upper airway constriction sites. It is not usually recommended to treat OSA using LAUP (laser-assisted uvulopalatoplasty). Radiofrequency ablation (RFA) may be a choice for patients with OSA who are unable to tolerate or resist positive airway pressure therapy or who have found oral appliances unsatisfactory. Palatal implants may be advantageous for certain people with mild OSA who cannot tolerate or refuse positive airway pressure therapy or who are having trouble with oral appliances. Postoperative follow-up evaluations should include objective assessments of the degree of sleep disturbance in breathing, oxygen saturation, and clinical evaluation for symptoms that persist. Long-term monitoring is necessary to detect disease relapse [29]. A multilayer surgical technique is a prospect for OSA patients who are still unresponsive to drug therapy [30]. In addition, a large number of OSA sufferers are looking for CPAP alternatives. Surgical techniques provide an alternative when CPAP compliance issues exist without the need for acceptable compliance [31]. Following nasal medical procedures, patients could encounter more noteworthy alleviation in their daytime weariness and wheezing than in other, by and large, medical problems. As indicated by research, treating individuals with obstructive rest apnea and nasal blockage with nasal medical procedures is successful [32].

## Conclusions

The sleep problem known as obstructive sleep apnea has serious health repercussions. In conclusion, there is a direct connection between OSA and ear, nose, and throat health. Repeated episodes of upper airway blockage during sleep, which disrupt breathing patterns, are the hallmark of OSA. The obstruction frequently impacts the tonsils, adenoids, and soft palate. Narrowing of the airway can be caused by anatomical anomalies of the soft palate, such as an extended uvula, or an abundance of tissue, which contributes to OSA. To treat these problems, surgical procedures like UPPP may be carried out. Managing OSA often involves collaboration between sleep medicine specialists and otorhinolaryngology professionals to address both the underlying causes and symptoms, ultimately improving overall patient well-being. The review's overall goal is to deepen our comprehension of the otorhinolaryngological components of OSA and to support better patient care and outcomes in this particular area. We tried to summarise the most recent study findings and developments that are relevant to the diagnosis and treatment of OSA and determine prospective areas for new developments in OSA management within the field of otorhinolaryngology.
